# Weak Exchange Interactions in Multispin Systems: EPR Studies of Metalloporphyrins Decorated with {Cr_7_Ni} Rings

**DOI:** 10.1021/acs.inorgchem.4c01248

**Published:** 2024-06-28

**Authors:** Fabio Santanni, Edmund Little, Selena J. Lockyer, George F. S. Whitehead, Eric J. L. McInnes, Grigore A. Timco, Alice M. Bowen, Roberta Sessoli, Richard E. P. Winpenny

**Affiliations:** † Dipartimento di Chimica “Ugo Schiff” & INSTM Unit, Università̀ degli Studi di Firenze, Via della Lastruccia 3, I50019 Sesto Fiorentino (Firenze), Italy; ‡ Photon Science Institute and Department of Chemistry, University of Manchester, Oxford Road, Manchester M13 9PL, United Kingdom

## Abstract

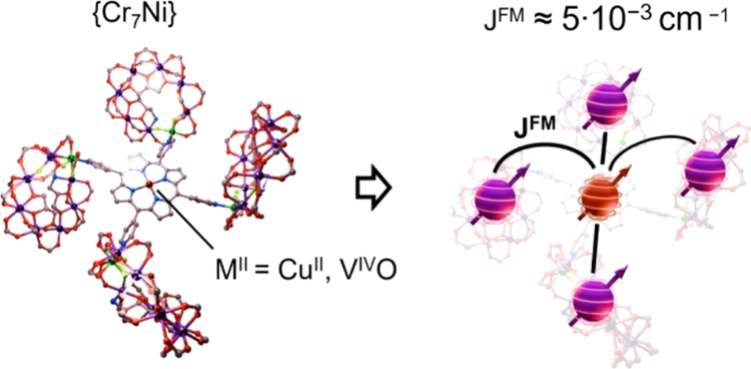

Both metalloporphyrins and heterometallic {Cr_7_Ni} rings are of significant research interest due to their proposed roles in quantum information processing devices. In this study, we present a series of complexes in which [Cr_7_NiF_3_(Etglu)(O_2_C*^t^*Bu)_15_] (*N*-EtgluH_5_ = *N*-ethyl-d*-*glucamine) heterometallic rings are coordinated to metalloporphyrin linkers: the symmetric [M(TPyP)] for M = Cu^2+^, VO^2+^, and H_2_TPyP = 5,10,15,20-tetra(4-pyridyl)porphyrin; and the asymmetric [{VO}(TrPPyP)] for H_2_(TrPPyP) = 5,10,15-(triphenyl)-20-(4-pyridyl)porphyrin. The magnetic interactions present in these complexes are unraveled using the continuous wave (CW) electron paramagnetic resonance (EPR) technique. The nature of the coupling between the {Cr_7_Ni} rings and the central metalloporphyrin is assessed by numerical simulations of CW EPR spectra and determined to be on the order of 0.01 cm^–1^, larger than the dipolar ones and suitable for individual spin addressability in multiqubit architectures.

## Abstract 2

The chemical and structural characterization of novel molecular adducts containing VO^2+^ and Cu^2+^-porphyrin complexes and one or four {Cr_7_Ni} magnetic rings is presented. A detailed investigation of static magnetic properties performed with continuous wave electron paramagnetic resonance has highlighted weak exchange coupling interactions between the central metalloporphyrin and the peripheral {Cr7Ni} rings, thus suitable for the realization of multiqubit spin architectures.

## Introduction

Qubits are the fundamental building blocks for any future quantum information processing (QIP).^1^ Much progress has been made in improving the performance of qubits based on electron spins in molecules,^2,3^ with coherence times reaching many microseconds, even at room temperature.^4−6^ For such qubits to be used to implement more complex logical operations than can be achieved with single-qubits, they need to be brought together into multiqubit architectures.^1,7^ Some work in this direction has been published, but much remains to be done, particularly in bringing together different qubits that could offer greater functionality than using the same qubit repeatedly.^7,8,9,10−18^ For example, qubits with different *g*-values could be addressed or measured separately.^10,12,19,20^

We are interested in investigating multiqubit architectures in which a central unit could switch the interaction between peripheral units.^15,20,21^ This might mimic a quantum circuit composed of several addressable qubits. A viable approach should satisfy three conditions: (i) we should use versatile molecular building blocks that help us in obtaining multiqubit architectures;^20,21^ (ii) we must control the interactions between the qubits such that they can be eventually used to generate entangled states;^20−23^ (iii) we must engineer a molecular structure in which all qubits are individually addressable because of different spin Hamiltonian parameters or different magnetic tensors orientation.^10−12,17−20^

The interaction between qubits must be sufficiently small to allow single-qubit operations as well as multiqubit interactions.^11,12^ In general, the size of this interaction should be smaller than the difference in the Zeeman energies of the two qubits, i.e.
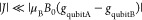
1but not too weak such that the splitting of the resonances should remain larger than the line broadening. As single spin addressability relies on the difference in the *g* values, here we study two potential qubits with *g-*values of 2.0 and 1.8, which imposes |*J*| to be less than 1 GHz (0.033 cm^–1^) in an X-band electron paramagnetic resonance (EPR) experiment and 3.4 GHz (0.1 cm^–1^) at Q-band.^24^ Such interactions are difficult to measure. Dipolar interactions can afford this size of interaction, but a more robust interaction with a narrower distribution of values can be achieved by increasing the distance between qubits while favoring superexchange interactions, e.g., using conjugated organic molecules.^10−12,17,18,24^ Ideally, one of our two qubits should involve a conjugated system.

These considerations have led us to look at a multispin architecture where one qubit is a metalloporphyrin and the second qubit is a {Cr_7_Ni} ring. We selected the metalloporphyrin for three reasons: (i) because of their versatility in attaching peripheral units including coordinating groups;^17,25−27^ (ii) the metal inside lays on a plane different to those of the external {Cr_7_Ni} mean planes, forcing the magnetic anisotropy tensor to be misaligned with respect to those of the second qubit (see below); (iii) the extended conjugation of π-bonds in porphyrins promotes appreciable superexchange coupling interactions from the pyrrolic core to the peripheries of the molecule.^11,12,18^ Indeed, exchange interactions in meso–meso-linked Cu^2+^ and VO^2+^ porphyrin dimers are in a good range for QIP and measurable with EPR spectroscopy.^11,12,18^

The {Cr_7_Ni} rings have long been studied as qubits and as components of multiqubit molecules.^13,15,16,20,28−34^ Here we have studied the chiral [Cr_7_NiF_3_(*N*-Etglu)(O_2_C*^t^*Bu)_15_L] rings (*N*-EtgluH_5_ = *N*-ethyl-d*-*glucamine; L = a monodentate ligand), which are purple in color.^25^ The monodentate ligand, L, is attached to the divalent Ni^2+^ center and can be displaced to link the purple rings. Moreover, {Cr_7_Ni} rings have unusual *g*-values of around 1.8 (in the *S* = 1/2 ground state) and are markedly anisotropic, allowing spectral addressability if oriented.

**Scheme 1:**
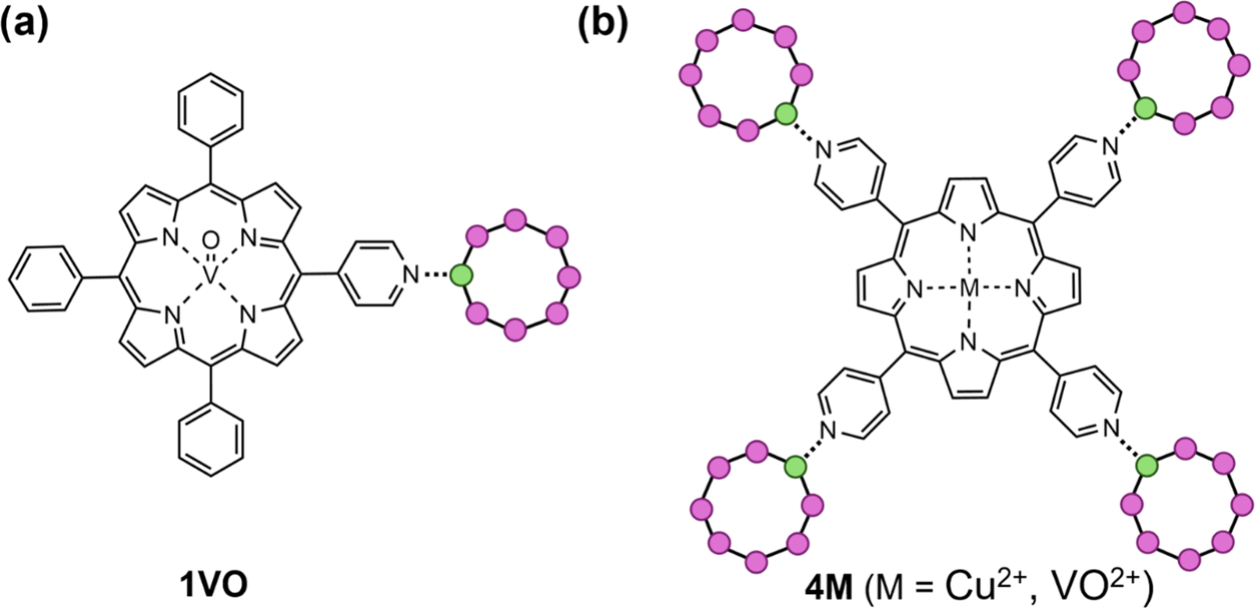
Schematic Representation of Molecular Structures for **1VO** (a) and **4M** (b) {Cr_7_Ni} rings are represented as circles (Cr = purple; Ni = green) superposed to an octagon.

Here, we have employed the VO^2+^ complex of monopyridyl-triphenyl (H_2_TrPPyP) porphyrin and the VO^2+^ and Cu^2+^complexes of tetrapyridyl porphyrin (H_2_TPyP) to obtain the three adducts of Scheme 1. Thanks to the meso-substituted pyridyl units, it is possible to coordinate a single {Cr_7_Ni} ring to [VO(TrPPyP)] giving {[Cr_7_NiF_3_(*N*-Etglu)(O_2_C*^t^*Bu)_15_][VO(TrPPyP)]} **1VO** (1 = one ring, VO = vanadyl-porphyrin) and four {Cr_7_Ni} rings to [M(TPyP)], giving {[Cr_7_NiF_3_(*N*-Etglu)(O_2_C*^t^*Bu)_15_]_4_[M(TPyP)]} (4 = four rings, M = VO^2+^ and Cu^2+^), **4VO** and **4Cu**. We then used EPR spectroscopy to study the magnetic coupling interactions through coordination bonds in these porphyrins, and we compared these interactions to those found in meso–meso-linked porphyrin systems.^11,12,18^

## Results and Discussion

### Synthesis and Structural Characterization

**Figure 1:**
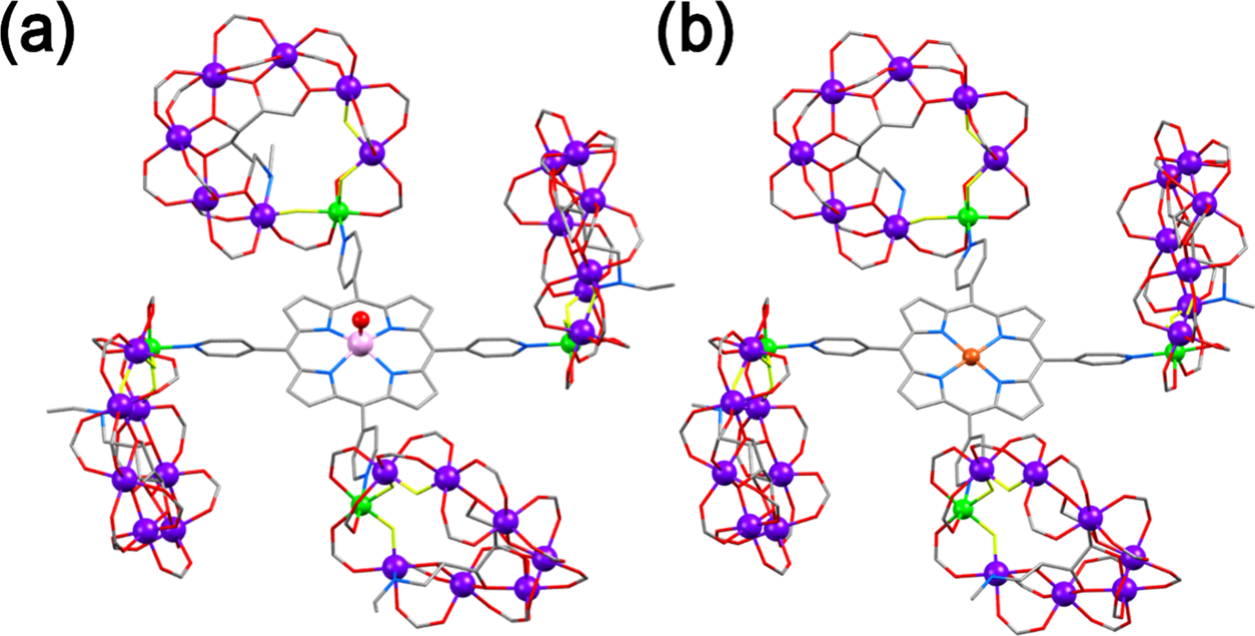
Simplified crystal structures of **4VO** (a) and **4Cu** (b). Color scheme: V, pink ball; Cu, orange ball; Ni, green balls; Cr, purple balls; O, red ball; N, blue sticks; C, gray sticks. H-atoms and methyl groups of ^*t*^BuCO_2_^–^ are omitted for clarity.

The complexes [VO(TrPPyP)], [M(TPyP)] and [Cr_7_NiF_3_(*N*-Etglu)(O_2_C*^t^*Bu)_15_(H_2_O)] were made by literature methods (see Section S1 of Supporting Information (SI)).^25,27,35,36^ Complexes **1VO**, **4VO**, and **4Cu** were then made by methods described in ref (25); it is worth stating that the low solubility of [M(TPyP)] is not a problem during the synthesis as the {Cr_7_Ni} ring solubilizes the porphyrin complex on coordination. Crystals of **4VO** and **4Cu** were grown, and the structures obtained from single crystal X-ray diffraction are shown in Figure 1 (see Figure S1 for the complete structures). The corresponding molecular packings are reported in Figures S2 and S3. Multiple attempts to crystallize **1VO** failed; it appears that, while four {Cr_7_Ni} rings give sufficient solubility to the porphyrin to allow crystals to grow, the solubility is insufficient if only one ring is present. Crystallographic parameters are given in Table S1.

In both **4VO** and **4Cu**, the nickel site of the {Cr_7_Ni} ring is bound to the pyridyl donor(s) from the porphyrin. The Ni–N bonds are between 2.038(16) and 2.090(14) Å. In **4M**, the angle between the porphyrin mean plane and the mean plane of each of the rings ranges from 80.94(11) to 115.91(11)° in **4VO** and 90.37(8) to 118.60(8)° in **4Cu**. This results in the rings being canted with respect to each other in **4M**: these angles are given in Table 1.

**Table 1:** Angles (deg) between Mean Planes of the Porphyrin (P) and Ring (R) Systems in **4VO** and **4Cu**

	**4VO**	**4Cu**
P–R	94.22(3), 75.31(3), 106.17(3), 117.42(3)	90.37(8), 92.78(8), 109.09(8), 118.60(8)
R–R, cis	106.46(4), 71.97(4), 62.53(4), 89.48(4)	94.22(3), 75.31(3), 106.17(3), 117.42(3)
R–R, trans	106.46(4), 71.97(4), 62.53(4), 89.48(4)	90.64(11), 97.83(10), 105.75(11), 115.91(11)

### Continuous Wave EPR (CW-EPR) Spectroscopy

**Figure 2:**
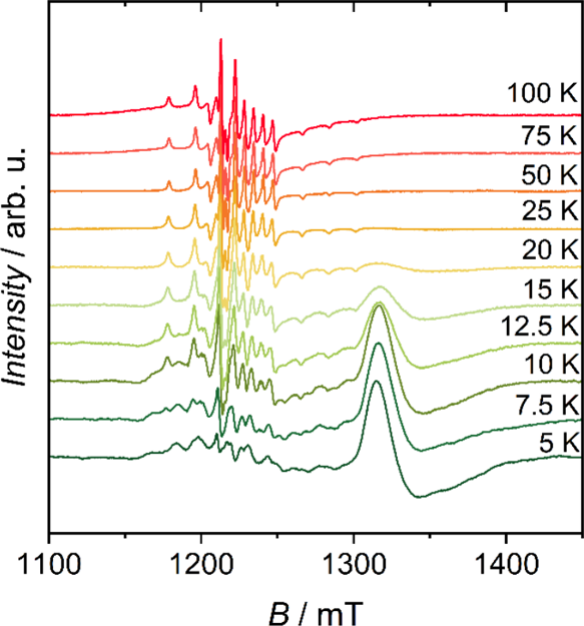
Variable temperature experimental Q-band CW-EPR spectra of **1VO**.

The variable temperature experimental Q-band spectra for **1VO** are shown in Figure 2. The hyperfine pattern typical of vanadyl-porphyrin (^51^V; natural abundancy—NA = 99.75%; *I* = 7/2) is seen at 100 K and centered at around *g* = 1.98.^11,35^ As the temperature is lowered, this pattern broadens slightly, and at around 20 K a broad resonance appears at *g* = 1.8, which is typical of a {Cr_7_Ni} ring.^25,31,34^ At lower temperatures, the *g* = 1.83 resonance becomes dominant in the spectra, with the vanadyl resonance lines broadening further. This is particularly noticeable below 12.5 K, where a splitting of most resonances becomes detectable.

To analyze these spectra, we begin with the simulation of the spectrum collected at 7.5 K. The dipolar coupling tensor was calculated considering point-dipole interaction between {Cr_7_Ni}–VO^2+^ placed at a mean (V···Ni) distance of 9.74(5) Å as calculated from the crystallographic structure of **4VO** (Figure S4).

**Figure 3:**
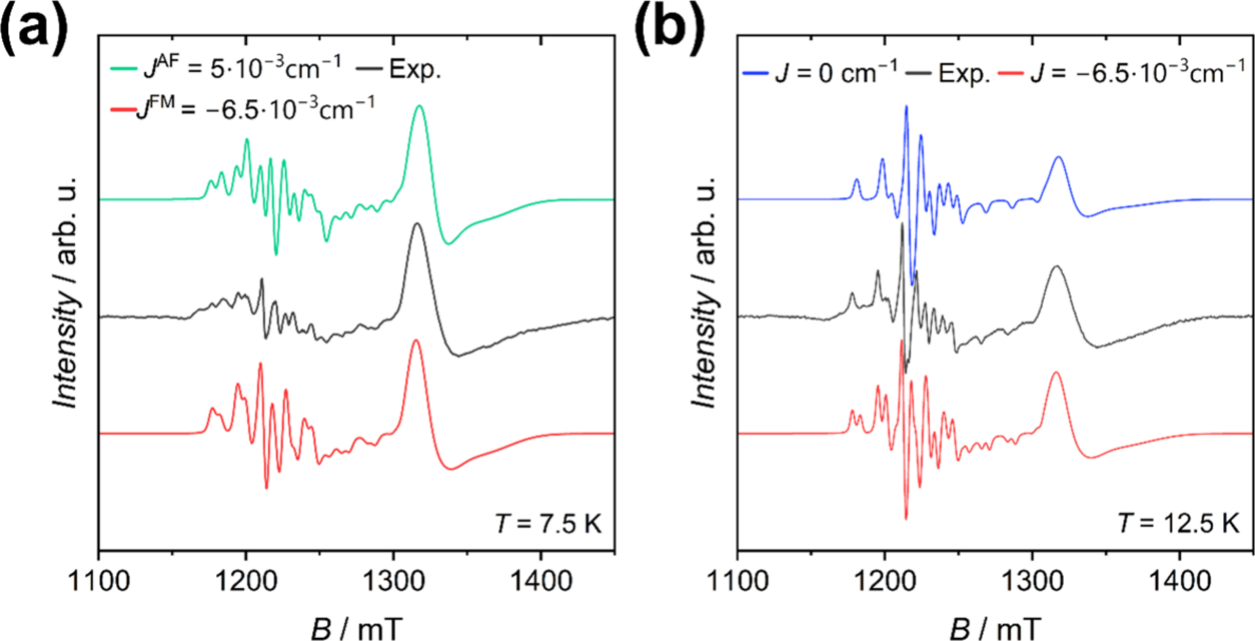
(a) Comparison between experimental and simulated EPR spectra of **1VO** at *T* = 7.5 K obtained by using the two possible values of *J*^FM^ = −6.5 × 10^–3^ cm^–1^ and *J*^AF^ = 5 × 10^–3^ cm^–1^. (b) Comparison between experimental and simulations of EPR spectra at 12.5 K with no exchange interaction (blue) or *J*^FM^ = −6.5 × 10^–3^ cm^–1^ interaction (red).

We then performed a series of simulations by varying the value of *J*, either considering ferromagnetic (FM) or antiferromagnetic (AF) exchange interactions (Figure 3a). All simulations used EASYSPIN (see also Section S3 of SI).^37^ The spin Hamiltonian employed for the simulations is
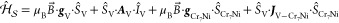
2where μ_B_ is the Bohr magneton, ***g***_V_ and ***g***_Cr_7_Ni_ are the *g*-tensors for VO^2+^ and {Cr_7_Ni} centers, respectively, ***A***_V_ is the hyperfine coupling interaction tensor for ^51^V. ***J***_V–Cr_7_Ni_ is the matrix describing the interaction contribution between the two centers.^38^ This includes both isotropic interaction (*J*) and through-space dipolar coupling (*D*), while it neglects the antisymmetric term.^38^
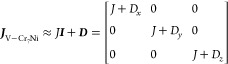
3Where the reference frame is that of the vanadyl porphyrin, i.e., *z* is the normal to the porphyrin plane, and *y* is the V–Ni direction. The dipolar tensor is computed considering the *g* anisotropy of both VO and {Cr_7_Ni}. However, off-diagonal terms of the *D* matrix are computed to be 3 orders of magnitude smaller than the diagonal ones and thus neglected in eq 3. The agreement with the experimental spectra was assessed on the basis of the magnitude of the splitting, which is a function of |*J*|, and the relative intensity of the peaks within the exchange-split doublets, which depends on the sign of *J*. The best agreement was found for a ferromagnetic (FM) exchange interaction of *J* = −6.5 × 10^–3^ cm^–1^, and other simulation parameters are reported in Table S2.

The sign is unambiguously determined, as can be seen in the comparison with the computed spectrum for antiferromagnetic (AF) exchange (see Figure 3a). The set of best-simulation parameters was hence employed as an initial guess to simulate all the spectra collected between 5 and 100 K (Figure S5 and Table S2). The temperature variation of *g*-strain parameters is reported in Figure S6.

Focusing on the spectrum recorded at 12.5 K, the simulation that includes the exchange interaction roughly reproduces the experimental spectrum, which instead shows features belonging to the simulations performed both with and without the exchange interaction (Figure 3b). This is particularly relevant for those VO^2+^ resonances between 1250 and 1300 mT. Above this temperature, the spectra are better reproduced by neglecting the interaction between the two spin units.

**Figure 4:**
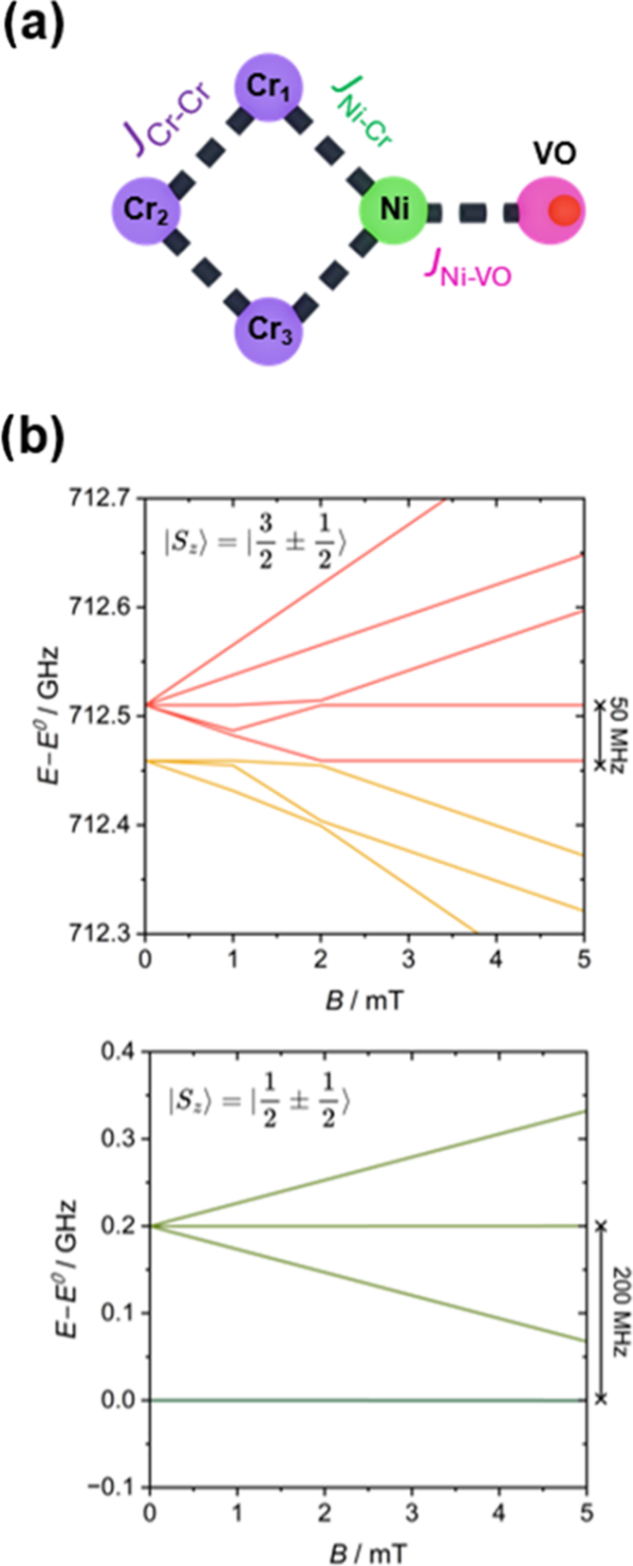
(a) Computed exchange model for the simplified system {Cr_3_Ni}–VO. *J*_Cr–Cr_ = 14 cm^–1^, *J*_Cr–Ni_ = 21 cm^–1^, *J*_Ni–VO_ = 0.01 cm^–1^. (b) Zoom of the Zeeman plots for the ground and first excited states.

We explain this behavior by considering the first excited spin state, *S* = 3/2, of the {Cr_7_Ni} ring, lies ca. 12 cm^–1^ above the ground state.^34^ Above 10 K this state is significantly populated and contributes to the spectrum. To support our hypothesis, we developed the following simplified multispin model to highlight the effect of the first excited spin manifold of the {Cr_7_Ni} ring. Other excited states were neglected as their energy is more than 30 cm^–1^ from the ground doublet.^34^ For ease of computation, we modeled the low energy spin states of the {Cr_7_Ni} ring using a {Cr_3_Ni} cluster (*g*^Cr^ = 2.0; *g*^Ni^ = 2.3) with the same isotropic exchange coupling values previously determined for the {Cr_7_Ni}^34^ and we introduced a weak coupling between the Ni spin to the vanadyl spin (*g*^VO^ = 1.98; *J* = 1 × 10^–2^ cm^–1^) as shown in Figure 4. The splitting induced by the weak exchange coupling with VO^2+^ on the *S* = 1/2 ground state of the {Cr_3_Ni} cluster is more significant than that involving the first excited state *S* = 3/2 (Figure 4). This means that the exchange-split EPR transitions of the VO^2+^ center when the {Cr_7_Ni} is in its excited *S* = 3/2 spin state are less separated than those involving the ground *S* = 1/2 ring state, and the effect of magnetic exchange is not visible within our experimental conditions. On lowering the temperature below 12.5 K, the population of *S* = 1/2 ground state of the ring cluster increases, thus enhancing the intensity of the exchange-split resonances in the VO^2+^ region. Therefore, the temperature variation in spectra is accounted for from the ladder of the spin states in {Cr_7_Ni} ring. Interestingly, also the *g*-strain parameters for VO^2+^ increase significantly below 15 K (Figure S6), suggesting that the selective population of the ground spin state of {Cr_7_Ni} also affects the line width, most likely through a more pronounced effect of the distribution in the ***J***_V–Cr_7_Ni_ values.

**Figure 5:**
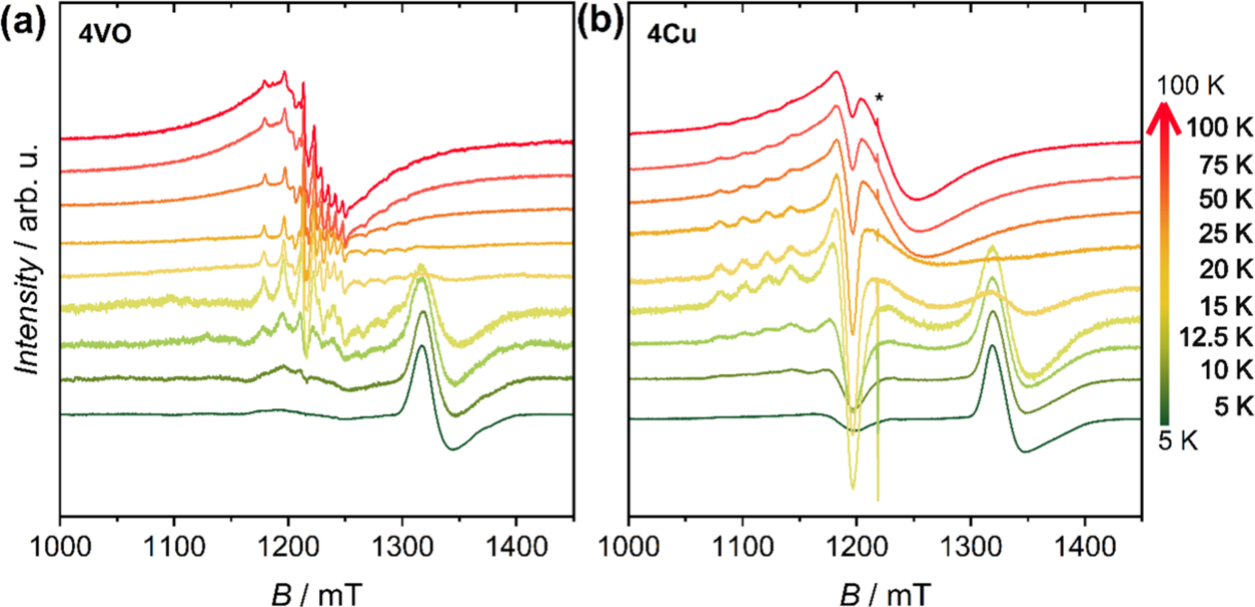
Temperature dependence of the Q-band EPR spectra of compounds: (a) **4VO** and (b) **4Cu** in 1.0 mM frozen solutions of 1:1 CH_2_Cl_2_/toluene between 5 and 100 K. An asterisk is placed in correspondence of a radical impurity commonly observed in porphyrins and phthalocyanines.^39^

Similar temperature variations are seen in **4VO** and **4Cu**. In **4VO** at 100 K, the frozen solution EPR spectrum shows the sharp resonances of vanadyl porphyrin complex (Figure 5a). Similar spectra, though with slightly broadened features, are observed in undiluted polycrystalline powders (Figure S7a), confirming the structure integrity in solution. In Figure 5 an underlying broad resonance due to the {Cr_7_Ni} ring is visible; this broad resonance is less pronounced in **1VO** as there is only one ring per vanadyl. As the temperature decreases below 20 K, a feature at *g* = 1.8 gains intensity, and the vanadyl resonances broaden significantly such that at 5 K the hyperfine interaction is almost completely unresolved. As in **1VO** the *g* = 1.8 resonance is due to the *S* = 1/2 ground state of the {Cr_7_Ni} ring.

For **4Cu** (frozen solution (Figure 5b), polycrystalline (Figure S7b)), at 100 K the broad high temperature resonance of the {Cr_7_Ni} rings dominates the broad resonances of the copper porphyrin. Cooling to 25 K reveals a distinctive copper porphyrin spectrum, with a strong resonance at *g* ≈ 2.05 and four associated resonances at lower field split by hyperfine coupling to the *I* = 3/2 ^63^Cu and ^65^Cu nuclei.^24^ As was observed for **4VO**, when the temperature is decreased further, the copper spectrum loses resolution as a resonance at *g* = 1.8 associated with the *S* = 1/2 ground state of {Cr_7_Ni} becomes more intense.

**Figure 6:**
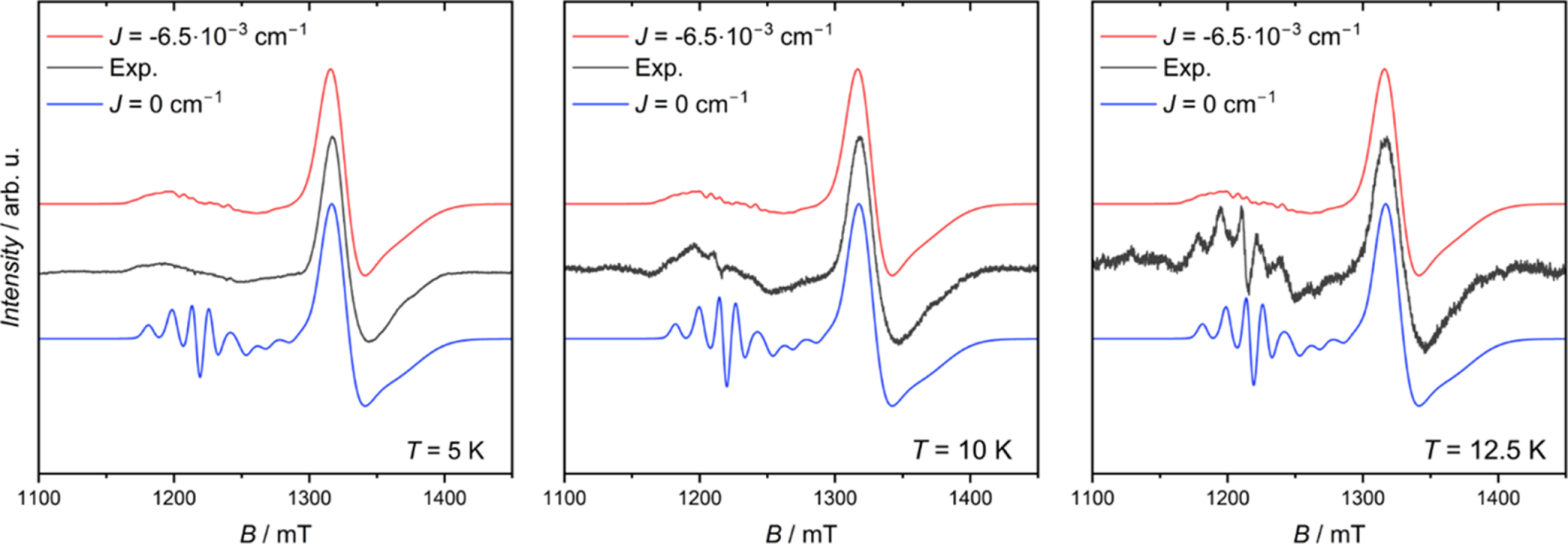
Plot of experimental and simulated Q-band EPR spectra of **4VO** 0.5 mM solutions in 1:1 CH_2_Cl_2_/toluene between 5 and 12.5 K.

As in **1VO** the broadening of the metalloporphyrin resonances at low temperatures implies an interaction between the central metalloporphyrin and the peripheral {Cr_7_Ni} clusters. To simulate the spectra for **4VO** we used the calculated dipolar interactions between the central metal and the Ni^2+^ in the rings, between Ni^2+^ ions on opposite rings, and between neighboring Ni^2+^ ions in the cross-like structure. The exchange interaction was fixed to the *J-*value found for **1VO** (Table 2). We also refined *g*-strain values, and the results highlight stronger broadening effects in **4VO** than in **1VO** (see Table S3 for all parameters). The increase in the strain can be attributed to the larger numbers of {Cr_7_Ni} rings attached to the porphyrin core, giving a larger distribution of interaction energies. Using these parameters, simulations reported in Figure 6 compare well with experimental data at low temperatures, but significant discrepancies are observed for **4VO** at 12.5 K, where a better agreement is obtained by neglecting the exchange interaction. As for **1VO** we attribute this to the reduced effect of VO^2+^–Ni^2+^ exchange interaction as the *S* = 3/2 state of the {Cr_7_Ni} ring is populated.

**Table 2:** Calculated Dipolar (*D_x_*_,_*_y_*_,_*_z_*) and Simulated Exchange Interactions (*J*) for the EPR Spectra of **1VO**, **4VO**, and **4Cu**

	M_i_–M*_j_*	*D*_*x*_^a^	*D*_*y*_^a^	*D*_*z*_^a^	*J*_iso_^b^
**1VO**	VO–Ni	1.7	–3.4	1.7	–6.5
**4VO**	VO–Ni_1_^c^	1.7	–3.4	1.7	–6.5
	Ni_1_–Ni_2_^d^	–2.2	1.1	1.1	0
	Ni_1_–Ni_3_^d^	0.19	–0.38	0.19	0
	Ni_1_–Ni_4_^d^	–2.2	1.1	1.1	0
**4Cu**	Cu–Ni_1_^c^	1.8	–3.6	1.8	<10^–2 ^e
	Ni_1_–Ni_2_^d^	–2.2	1.1	1.1	0
	Ni_1_–Ni_3_^d^	0.19	–0.38	0.19	0
	Ni_1_–Ni_4_^d^	–2.2	1.1	1.1	0

a |*D*_*x*,*y*,*z*_| = 10^–3^ cm^–1^.

b |*J*| = 10^–3^ cm^–1^.

c **D** components for the other M–Ni*_i_* (M = Cu, VO, *i* = 2, 3, 4) are omitted for simplicity.

d All the other permutation of N_i_–N*_j_* couples have been considered as shown in EASYSPIN codes in Section S4.

e Absolute value of *J*.

Passing to **4Cu**, which presents less resolved spectra, we performed a survey of computed spectra with different values of *J* and of *g*-strain (see Figures S8–S10). These allowed us to estimate that the exchange interaction has a similar order of magnitude as in **4VO**, with an upper limit of 1 × 10^–2^ cm^–1^. Also in this case, a better agreement with the experimental spectra recorded at higher temperatures is achieved by fixing *J* to zero (Figure S11 and Table S4).

## Conclusions

Three examples of metalloporphyrins decorated with chiral {Cr_7_Ni} rings have been synthesized, and EPR spectroscopy has been used to measure the interaction energies between the units in the structure in an energy range that is inaccessible to conventional magnetometry. Continuous wave EPR spectroscopy indicates the interaction between the metalloporphyrin and the ring is around −0.0065 cm^–1^ (FM) for vanadyl porphyrins, and of comparable strength for copper porphyrins. This similarity could be surprising, given the weaker σ overlap between the d_*xy*_ magnetic orbital of the vanadyl ion and those of the porphyrin scaffold compared to the Cu^II^ case. However, the capability of the vanadyl unit to promote sizable exchange interactions was already observed in meso–meso linked homo and heterometallic porphyrin dimers.^11,12,18^ An exchange pathway was identified in the overlap of the porphyrin out-of-plane orbitals with the metal magnetic orbital because the vanadium ion is not sitting on the plane of the porphyrin. Our findings reinforce previous observations on the versatility of metal porphyrins and underscore the remarkable potential of the vanadyl ion in facilitating suitable coupling interactions to realize multispin qubit architectures.

## Supporting Information Available

Additional information about the synthesis and characterization (spectroscopy and X-ray diffraction) of compounds (Sections S1 and S2); additional EPR spectra and EASYSPIN codes (Sections S3 and S4, respectively) (PDF)
