# Supplementary material for: Weak Exchange Interactions in Multispin Systems: EPR Studies of Metalloporphyrins Decorated with {Cr7Ni} Rings
Source: Inorg Chem. 2024 Jun 28;63(33):15460–6. doi: 10.1021/acs.inorgchem.4c01248 (PMC11337161; doi:10.1021/acs.inorgchem.4c01248)
Supplement: Supplementary file 1 — ic4c01248_si_001.pdf [file ic4c01248_si_001.pdf]

Weak Exchange Interactions in Multispin Systems: EPR Studies of Metalloporphyrins Decorated with $\{Cr_7Ni\}$ Rings

Fabio Santanni,[1] Edmund Little,[2] Selena J. Lockyer,[2] George F. S. Whitehead,[2] Eric J. L. McInnes,[2] Grigore A. Timco,[2] Alice Bowen,[2] Roberta Sessoli[1]* Richard E. P. Winpenny[2]*

[1]*Dipartimento di Chimica "Ugo Schiff", Università degli Studi di Firenze, Via della Lastruccia 3, I50019 Sesto, Fiorentino (Firenze), Italy*

[2]*Photon Science Institute and Department of Chemistry, University of Manchester, Oxford Road, Manchester, M13 9PL, United Kingdom*

Electronic Supporting Information

<u>Corresponding Authors</u>

* Roberta Sessoli – Email: roberta.sessoli@unifi.it

* Richard E. P. Winpenny – Email: richard.winpenny@manchester.ac.uk

S.1 Synthetic procedures

All chemicals were of reagent grade and used without further purification. Ligands H_2TPyP and $H_2TrPPyP$ were purchased from Sigma-Aldrich and Porphychem Sas, respectively. VO^{2+} and Cu^{2+} complexes – [VO(TrPPyP)], [VO(TPyP)], and [Cu(TPyP)] - were synthesized by modification of the reported procedures.[1,2,3] The syntheses of **1VO** and **4M** (M = VO, Cu) were performed by adopting the general procedure reported before.[4] All operations involving inert atmospheres were conducted under N_2 using standard Schlenk techniques. Reactions were followed by TLC.

[VO(TrPPyP)]

The synthesis was adapted from ref.[1]. $H_2TrPPyP$ (50 mg, 0.08 mmol) was added to PhOH (2 g), and the temperature was slowly increased to 80 °C while keeping the mixture under N_2. $[VO(acac)_2]$ ($acac^-$ = acetylacetonate, 33 mg, 0.12 mmol) was added to the stirred solution, and the temperature increased up to 165 °C. The reaction was left stirring under nitrogen for 13 hours. The product formation was confirmed via UV-Vis spectroscopy. Once reacted, PhOH was removed by vacuum distillation and subsequent heating of the crude product to 140 °C under flowing N_2 for 3 hours. The solid product obtained was dissolved in a minimum amount of EtOAc and purified by flash column chromatography on silica, adopting a mixture EtOAc/MeOH 95:5 as eluent. Yield: 22 mg, 40%. UV-Vis (DCM): λ max = 424 nm, 546 nm, 582 nm. FT-IR (KBr, cm^{-1}) = 3113(vw), 3018(vw), 1591(s), 1544(w), 1487(vw), 1440(w), 1412(m), 1327(m), 1261(w), 1205(m), 1165(vw), 1073(m), 1000(vs, V=O stretching), 874(vw), 809(s), 724(m, s), 658(s),583(m). MALDI-MS (m/z): 681.5 ([M+H^+]).

[VO(TrPPyP)(Cr_7NiF_3(Etglu)(O_2CtBu)$_{15}$)] (1VO)

The synthesis was adapted from ref.[4]. Compounds [VO(TrPPyP)] and [Cr_7NiF_3(Piv)$_{15}$(Etglu)(H_2O)] were combined in equimolar ratio in CH_2Cl_2 and stirred for 48h. Then, the solvent was removed, and the residue was washed with MeCN and dried. The obtained powder was dissolved in pentane and filtered. Upon slow evaporation of pentane, the overall procedure qualitatively yielded a red crystalline powder.
Elemental analysis (EA) found (calcd. for $C_{125}H_{174}Cr_7F_3NiN_6O_{36}V$): V, 1.13 (1.48); Cr 13.00 (12.69); Ni 2.12 (2.05); C, 49.70 (52.36); H, 6.42 (6.12); N, 2.40 (2.93).

[VO(TPyP)]

The synthesis was performed by modifying the procedure from ref.[2] H_2TPyP (1.1 g, 1.8 mmol) and $[VO(acac)_2]$ (650 mg, 2.45 mmol) were added to a DMF/CH_3COOH mixture (1:1, 160 mL) and refluxed under N_2. After 24 h, the reaction was stopped, and the solution was left to cool to room temperature. The reaction mixture was then poured onto 500 mL of water and ice and stirred for 30 minutes. The resulting mixture was filtered on a glass frit, and the collected purple powder (residual ligand) was washed several times with water (3 x 20 mL), acetone (4 mL), and Et_2O (10 mL). Thus, the red solution was extracted three times with chloroform (100 mL). All the fractions were collected,

dried over $MgSO_4$, and filtered. The solvent was removed under reduced pressure. The crude product was purified by column chromatography on flash silica gel using CH_2Cl_2/MeOH (96:4) as the eluent. The overall process yielded 653 mg of [VO(TPyP)]·$2H_2O$ (yield: 50%). The presence of water molecules was confirmed by elemental analysis. EA found (calcd. for $VC_{40}H_{28}N_8O_3$): V, 6.64 (7.08); C, 67.12 (66.76); H, 4.14 (3.92); N, 14.80 (15.57). UV-Vis (CH_2Cl_2): λmax = 421 nm, 544 nm, 580 nm. FT-IR (cm^{-1}) = 3111(vw), 3015(vw), 1592(s), 1547(w), 1408(m), 1333(m), 1201(w), 1068(w), 1000(vs, V=O stretching), 892(w), 852(vw), 802(s), 727 (s), 658 (s). MALDI-MS (m/z): 684.6 ([M+H+]).

General synthesis procedure for [M(TPyP)(Cr_7NiF_3(Etglu)(O_2C^tBu)$_{15}$)$_4$] (4M)

The syntheses of these compounds followed the general strategy reported in ref.[2] [M(TPyP)] and [Cr_7NiF_3(Piv)$_{15}$(Etglu)(H_2O)] were added to CH_2Cl_2 and stirred for 72 h at room temperature. The solvent was then removed under reduced pressure, and the crude product was dispersed in acetone and left to stir overnight. The mixture was filtered, and the obtained powder was washed several times with acetone. The crude product was extracted with pentane and filtered again. Acetone was added to the solution in 1:1 ratio. A crystalline powder precipitated from the solution upon slow evaporation of solvents.

[VO(TPyP)(Cr_7NiF_3(Etglu)(O_2C^tBu)$_{15}$)$_4$] (4VO).

The synthesis was performed by adopting the general procedure reported in ref.[4] [VO(TPyP)] (160 mg, 0.23 mmol) and [Cr_7NiF_3(Piv)$_{15}$(Etglu)(H_2O)] (2.2 g, 0.99 mmol) were reacted in 50 mL of a CH_2Cl_2 for three days. After the reaction and work-up of the reaction product, the overall process qualitatively yielded a red crystalline powder. X-ray quality crystals were obtained by slow diffusion of acetone's vapors into a hexane solution of **4VO**. EA found (calcd. for $C_{372}H_{648}N_{12}O_{145}VCr_{28}Ni_4$): V, 0.47 (0.53); Cr, 14.52 (15.80); Ni, 2.75 (2.53); C, 46.87 (46.65); H, 6.55 (6.48); N, 1.77 (1.75).

[Cu(TPyP)(Cr_7NiF_3(Etglu)(O_2C^tBu)$_{15}$)$_4$] (4Cu).

The synthesis was performed by adopting the general procedure reported in ref.[4] [Cu(TPyP)] (90 mg, 0.13 mmol) and [Cr_7NiF_3(Piv)$_{15}$(Etglu)(H_2O)] (1 g, 0.45 mmol) were reacted in 22 mL of a CH_2Cl_2/MeOH (10:1) mixture for three days. After the reaction and work-up of the product, the overall process qualitatively yielded a red crystalline powder. X-ray quality crystals were obtained by slow diffusion of acetone into a hexane solution of **4Cu**. EA found (calcd. for $C_{372}H_{648}N_{12}O_{145}CuCr_{28}Ni_4$): Cr 14.82 (15.55); Cu, 0.70 (0.68); Ni, 2.72 (2.51); C, 47.61(47.72); H, 6.84 (6.98); N, 1.89 (1.80).

S.2 Structural characterization

Data collection

X-ray diffraction data for compounds **4VO** and **4Cu** were collected using a dual-wavelength Rigaku FR-X rotating anode diffractometer using CuKα (λ = 1.54146 Å) radiation, equipped with an AFC-11 4-circle goniometer, VariMAX^{TM} microfocus optics, a Hypix-6000HE detector and an Oxford Cryosystems 800 plus nitrogen flow gas system, at a temperature of 150K and 100K, respectively. Data were collected and reduced using CrysAlisPro v42.[5] Absorption correction was performed using empirical methods (SCALE3 ABSPACK) based upon symmetry-equivalent reflections combined with measurements at different azimuthal angles.

Crystal structure determination and refinements.

The crystal structure was solved and refined against all F^2 values using the SHELX and Olex2 suite of programs.[6-8] Coordinates for all nonhydrogen atoms were freely refined, and atomic displacement parameters were refined anisotropically. Hydrogen atom coordinates and isotropic atomic displacement parameters were constrained to ride on the coordinates and atomic displacement parameters of the parent atom. Global similar neighbor atomic displacement parameter and enhanced rigid bond restraints were applied globally, and strong similar neighbor atomic displacement parameter restraints were applied to all carbon atoms. These were applied to improve and refine realistic atomic displacement parameters, given the low resolution and limited number of data points for the dataset and the statistical disorder of the methyl groups of the pivalate moieties, which could not be modeled due to the limited number of data points and a requirement to not over-parameterize the model. Similar pivalate moieties were refined to have similar 1,2- and 1,3- bond distances, and where necessary when modeling solvent molecules, fixed distance restraints were applied.

Please note: For **4VO**, the oxygen of the vanadyl has been modeled over two positions but 100% oxygen; this is because 50% of the time, this is a water hydrogen bonding to the opposite face of the porphyrin to the slightly out-of-plane vanadyl. This is the only explanation that could be rationalized for the electron density present at that position.

Table S1. Experimental and refinement parameters extracted from the resolution of crystallographic structures of compounds 4VO and 4Cu.

	**4VO**	**4Cu**
Empirical formula	$C_{375}H_{634}Cr_{28}F_{12}N_{12}Ni_4O_{145}V$	$C_{491}Cr_{28}CuF_{12}H_{756}N_{21}Ni_4O_{140}$
Formula weight	9600.68	11175.5
Temperature/K	150.00(11)	99.97(12)
Crystal system	monoclinic	monoclinic
Space group	$P2_1$	$P2_1$
a/Å	31.7977(12)	31.7215(5)
b/Å	28.2327(6)	30.3949(4)
c/Å	32.8855(12)	32.2482(6)
α/°	90	90
β/°	112.830(4)	109.648(2)
γ/°	90	90
Volume/Å^3	27209.7(17)	29282.5(9)
Z	2	2
ρ_{calc}g/cm^3	1.172	1.267
μ/mm^{-1}	5.294	4.902
F(000)	10086	11780
Crystal size/mm^3	0.3 × 0.199 × 0.029	0.556 × 0.516 × 0.347
Radiation	Cu Kα (λ = 1.54184)	Cu Kα (λ = 1.54184)
2Θ range for data collection/°	4.278 to 90.332	2.958 to 86.994
Index ranges	-29 ≤ h ≤ 28, -24 ≤ k ≤ 25, -30 ≤ l ≤ 30	-28 ≤ h ≤ 28, -27 ≤ k ≤ 27, -28 ≤ l ≤ 28
Reflections collected	111362	271574
Independent reflections	41122 [R_{int} = 0.0834, R_{sigma} = 0.1175]	43652 [R_{int} = 0.0881, R_{sigma} = 0.0515]
Data/restraints/parameters	41122/37030/5158	43652/36276/5271
Goodness-of-fit on F^2	1.063	1.033
Final R indexes [I>=2σ (I)]	R_1 = 0.0725, wR_2 = 0.1826	R_1 = 0.0638, wR_2 = 0.1710
Final R indexes [all data]	R_1 = 0.1193, wR_2 = 0.2055	R_1 = 0.0676, wR_2 = 0.1745
Largest diff. peak/hole / e Å^{-3}	0.55/-0.41	0.53/-0.39
Flack parameter	0.003(4)	0.031(2)

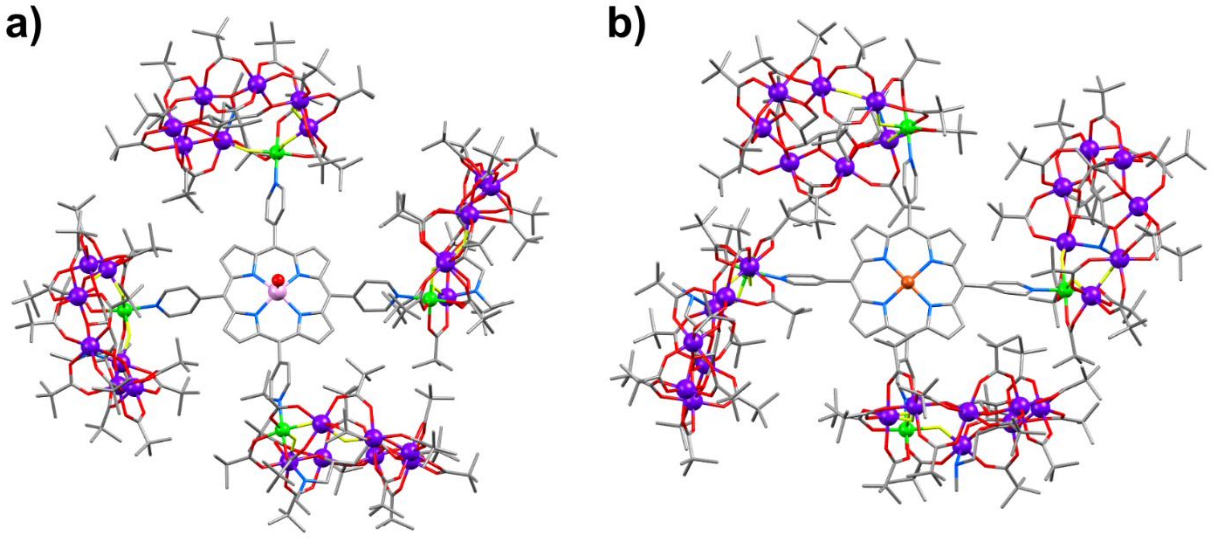

Figure S1. Molecular structure of a) **4VO** and b) **4Cu**. Solvent molecules and hydrogen atoms have been omitted for clarity. Color scheme: V = pink, Cu = orange, Cr = purple, Ni = green, O = red, N = blue, C = grey; *a* = red, *b* = green, *c* = blue.

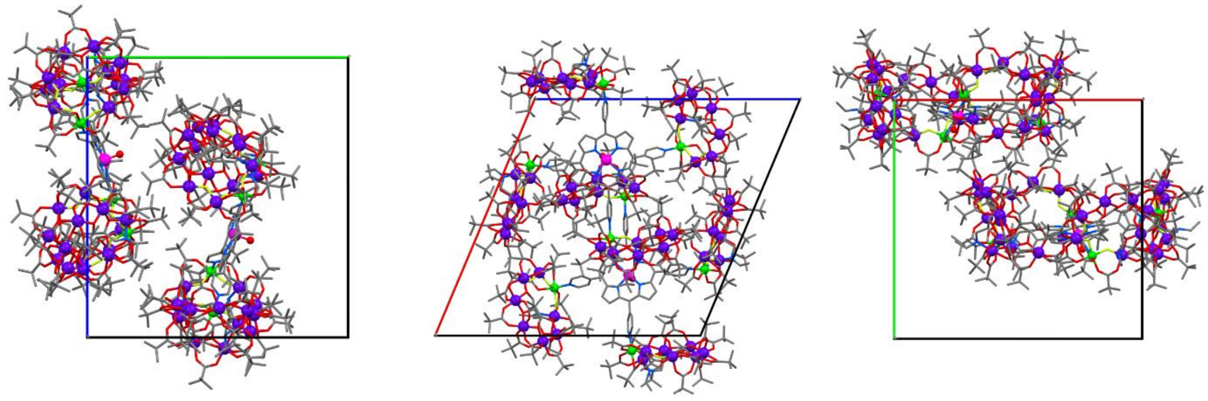

Figure S2. Crystallographic structure of **4VO**. From the left to the right, view of the unit cell along the *a*, *b*, and *c* crystallographic axes. Color scheme: V = pink, Cr = purple, Ni = green, O = red, N = blue, C = grey; *a* = red, *b* = green, *c* = blue. H atoms have been omitted for clarity.

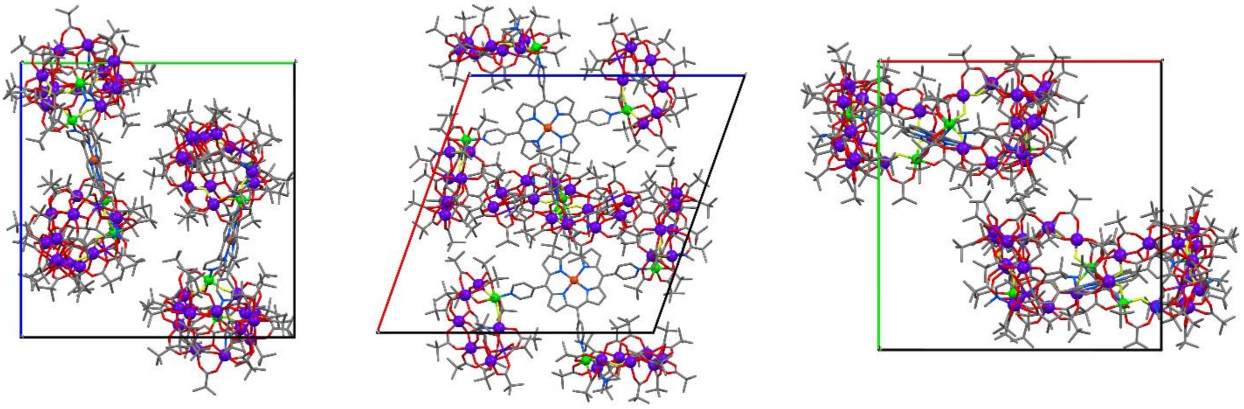

Figure S3. Crystallographic structure of **4Cu**. From the left to the right, view of the unit cell along the *a*, *b*, and *c* crystallographic axes. Color scheme: Cu = orange, Cr = purple, Ni = green, O = red, N = blue, C = grey; *a* = red, *b* = green, *c* = blue. H atoms have been omitted for clarity.

S.3 CW-EPR spectroscopy

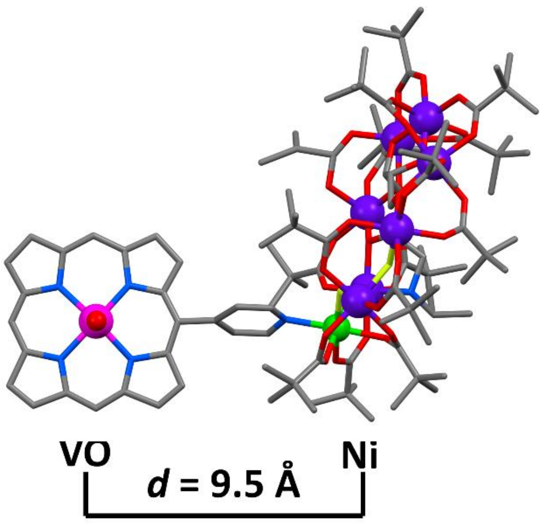

	VO-Ni
D_x (cm^{-1})	0.0017
D_y (cm^{-1})	-0.0034
D_z (cm^{-1})	0.0017
J_{iso} (cm^{-1})	0.0065

Figure S4. On the left, hypothetic simplified molecular structure of **1VO**. Color scheme: V = pink, Cr = purple, Ni = green, O = red, N = blue, C = grey. On the right, table reporting **D** components computed for VO-Ni placed at a mean distance *d*, and J_{iso} from best simulations.

Table S2. Best-simulation parameters for **1VO**. [a] 10^{-4} cm^{-1}. [b] gStrain (FWHM) components.

	T(K)	g_x	g_y	g_z	A_x[a]	A_y[a]	A_z[a]	Γ_x[b] (10^{-3})	Γ_y[b] (10^{-3})	Γ_z[b] (10^{-3})
**VO**	5	1.9790(1)	1.9790(1)	1.9580(1)	56(2)	56(2)	161(2)	10(1)	10(1)	9.0(5)
	7.5	1.9790(1)	1.9790(1)	1.9580(1)	56(2)	56(2)	161(2)	7.5(5)	7.5(5)	6.5(5)
	10	1.9790(1)	1.9790(1)	1.9580(1)	56(2)	56(2)	161(2)	6.5(5)	6.5(5)	6.0(5)
	12.5	1.9790(1)	1.9790(1)	1.9580(1)	56(2)	56(2)	161(2)	4.5(5)	4.5(5)	3.5(5)
	15	1.9842(1)	1.9842(1)	1.9631(1)	56(2)	56(2)	161(2)	3.5(5)	3.5(5)	1.7(2)
	20	1.9842(1)	1.9842(1)	1.9631(1)	56(2)	56(2)	161(2)	2.5(5)	2.5(5)	1.0(5)
	25	1.9842(1)	1.9842(1)	1.9631(1)	56(2)	56(2)	161(2)	2.5(5)	2.5(5)	1.0(5)
	50	1.9842(1)	1.9842(1)	1.9631(1)	56(2)	56(2)	161(2)			
	75	1.9842(1)	1.9842(1)	1.9631(1)	56(2)	56(2)	161(2)			
	100	1.9842(1)	1.9842(1)	1.9631(1)	56(2)	56(2)	161(2)			
**Cr_7Ni**	5	1.843(1)	1.830(1)	1.778(1)				15(1)	20(1)	50(2)
	7.5	1.843(1)	1.830(1)	1.778(1)				15(1)	25(1)	55(2)
	10	1.843(1)	1.830(1)	1.778(1)				15(1)	25(1)	60(2)
	12.5	1.843(1)	1.830(1)	1.778(1)				15(1)	25(1)	60(2)
	15	1.841(1)	1.828(1)	1.777(1)				16(1)	39(2)	65(2)

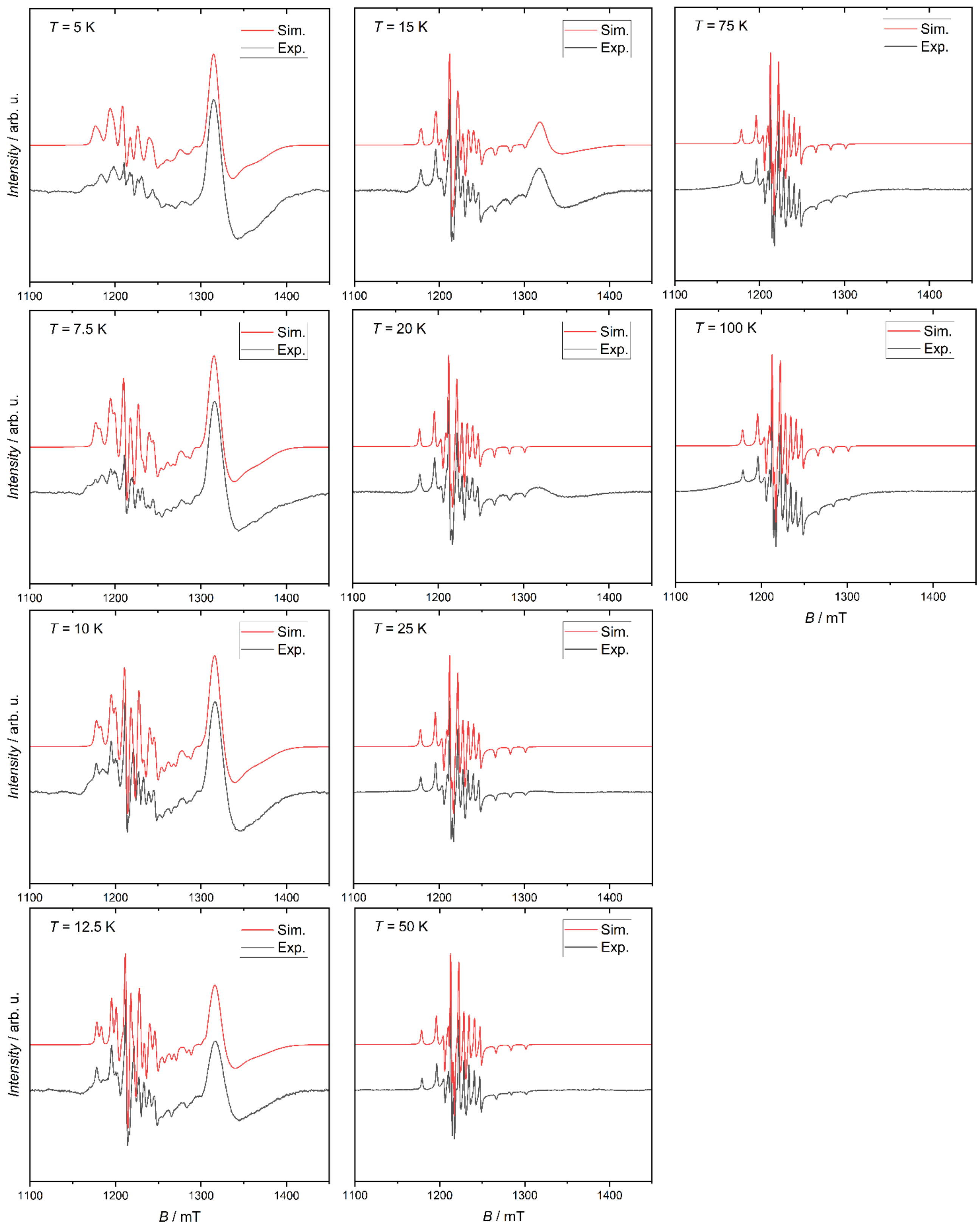

Figure S5. Plot of all experimental Q-band CW-EPR spectra collected on **1VO** 0.5 mM solution in 1:1 CH_2Cl_2/toluene at different temperatures and their simulations obtained using parameters in Table S2.

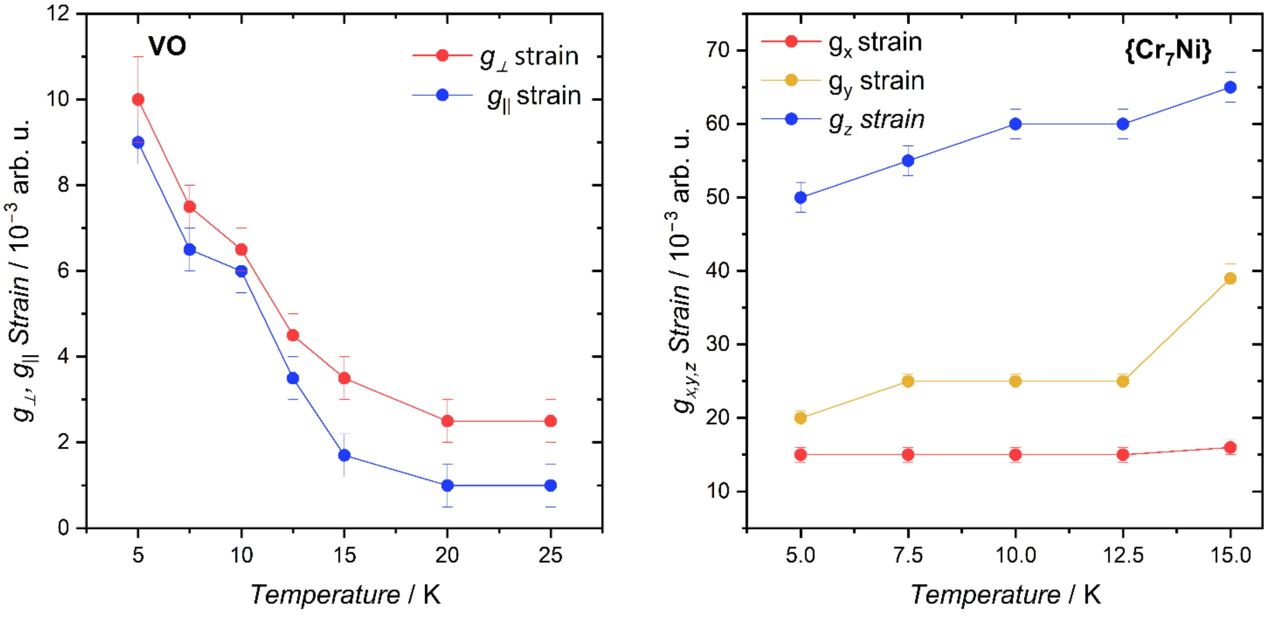

Figure S6. On the left, plot of g-strain components ($g_x = g_y = g_\perp$; $g_z = g_{||}$) variation as a function of T for VO spin in **1VO**. On the right, plot of g-strain components variation as a function of T for the {Cr_7Ni} ring in **1VO**.

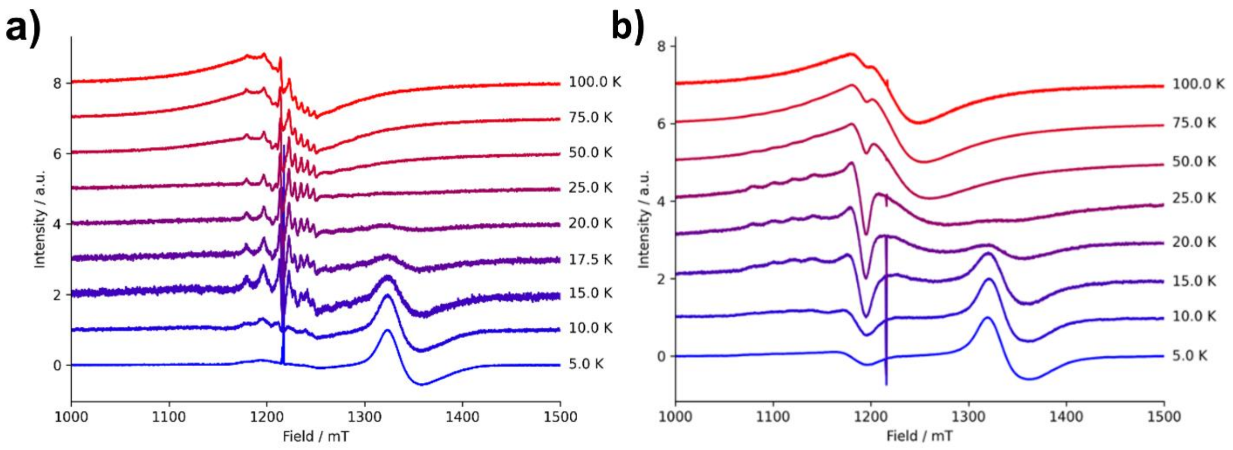

Figure S7. Temperature dependence of the polycrystalline Q-band EPR spectra of (a) **4VO** and (b) **4Cu** between 5 K and 100 K.

Table S3. Best-simulation SH parameters for **4VO**. [a]10^{-4} cm^{-1}. [b] g-strain (FWHM) components.

	T(K)	g_x	g_y	g_z	A_x[a]	A_y[a]	A_z[a]	Γ_x[b] (10^{-3})	Γ_y[b] (10^{-3})	Γ_z[b] (10^{-3})
**VO**	5	1.9790(1)	1.9790(1)	1.9580(1)	56(2)	56(2)	161(2)	10(1)	10(1)	9.0(5)
	10	1.9790(1)	1.9790(1)	1.9580(1)	56(2)	56(2)	161(2)	9.5(5)	9.5(5)	7.5(5)
	12.5	1.9790(1)	1.9790(1)	1.9580(1)	56(2)	56(2)	161(2)	9.5(5)	9.5(5)	7.5(5)
**Cr_7Ni**	5	1.843(1)	1.830(1)	1.778(1)				25(1)	30(1)	60(2)
	10	1.843(1)	1.830(1)	1.778(1)				25(1)	30(1)	60(2)
	12.5	1.843(1)	1.830(1)	1.778(1)				25(1)	30(1)	60(2)

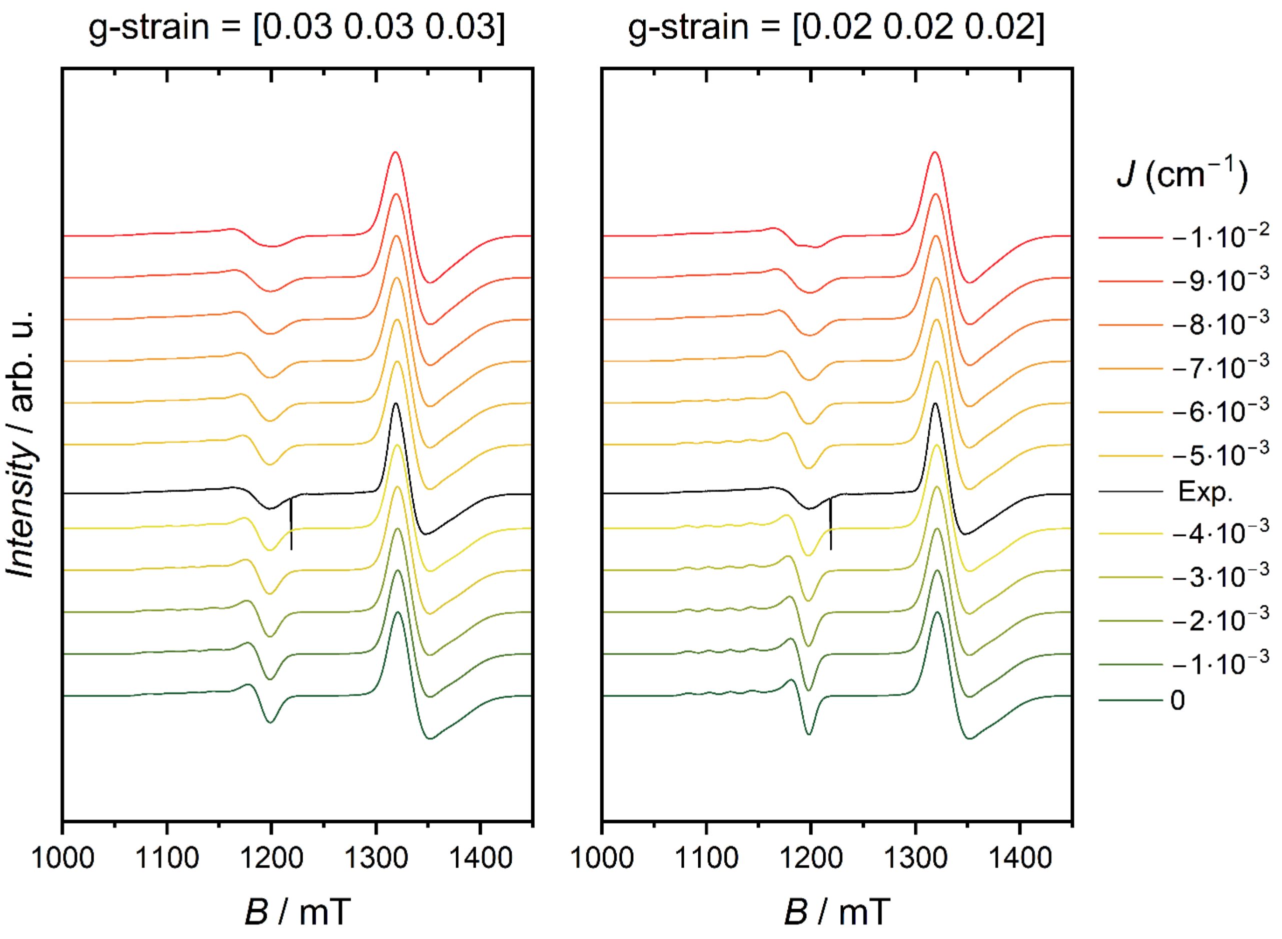

Figure S8. Plot of experimental and simulated Q-band EPR spectra of **4Cu** 0.5mM solutions in 1:1 CH_2Cl_2/toluene at 5 K. Simulations were performed for two different sets of g-strain components by varying *J* (FM).

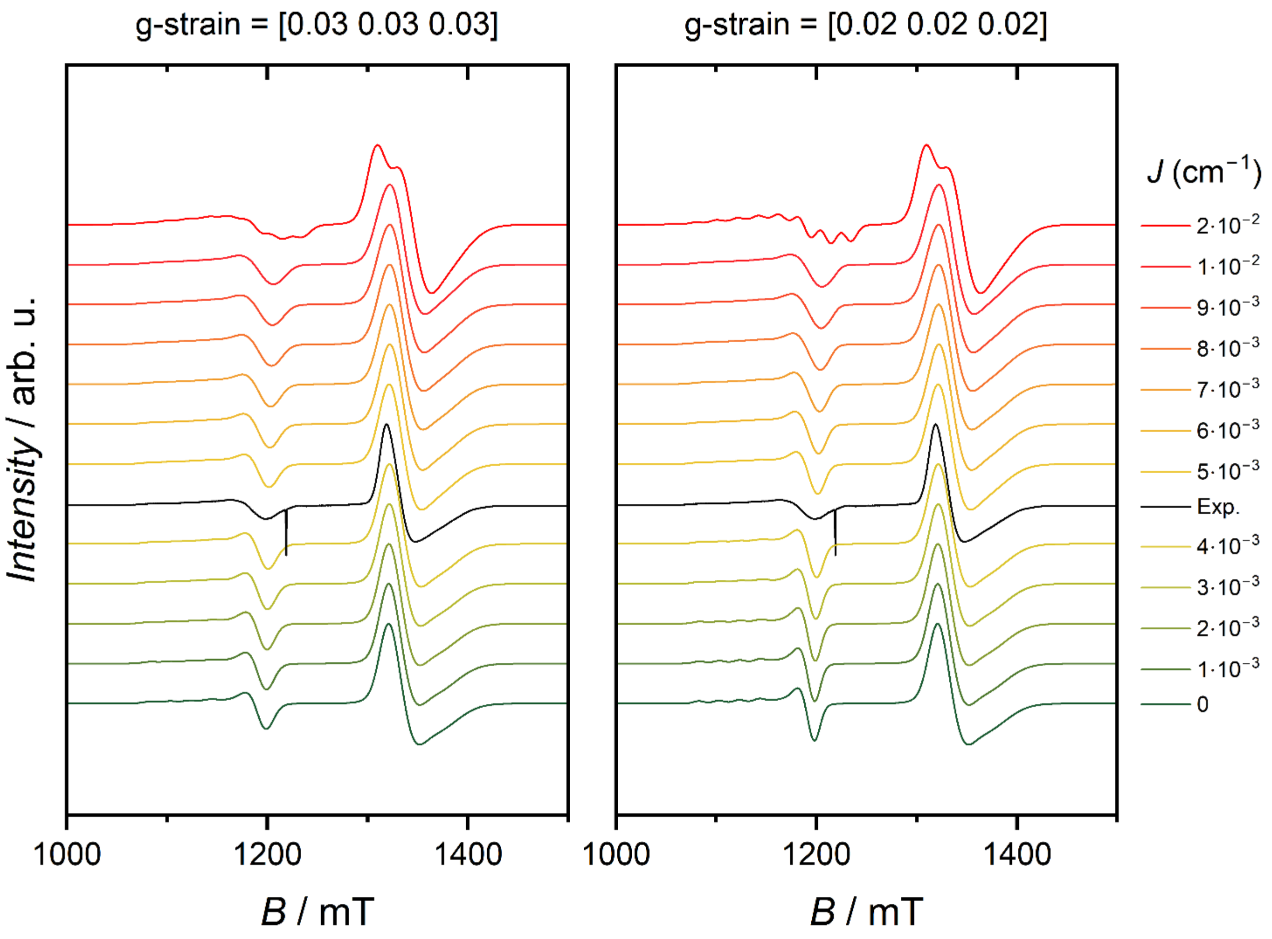

Figure S9. Plot of experimental and simulated Q-band EPR spectra of **4Cu** 0.5mM solutions in 1:1 CH_2Cl_2/toluene at 5 K. Simulations were performed for two different sets of g-strain components by varying *J* (AF).

.

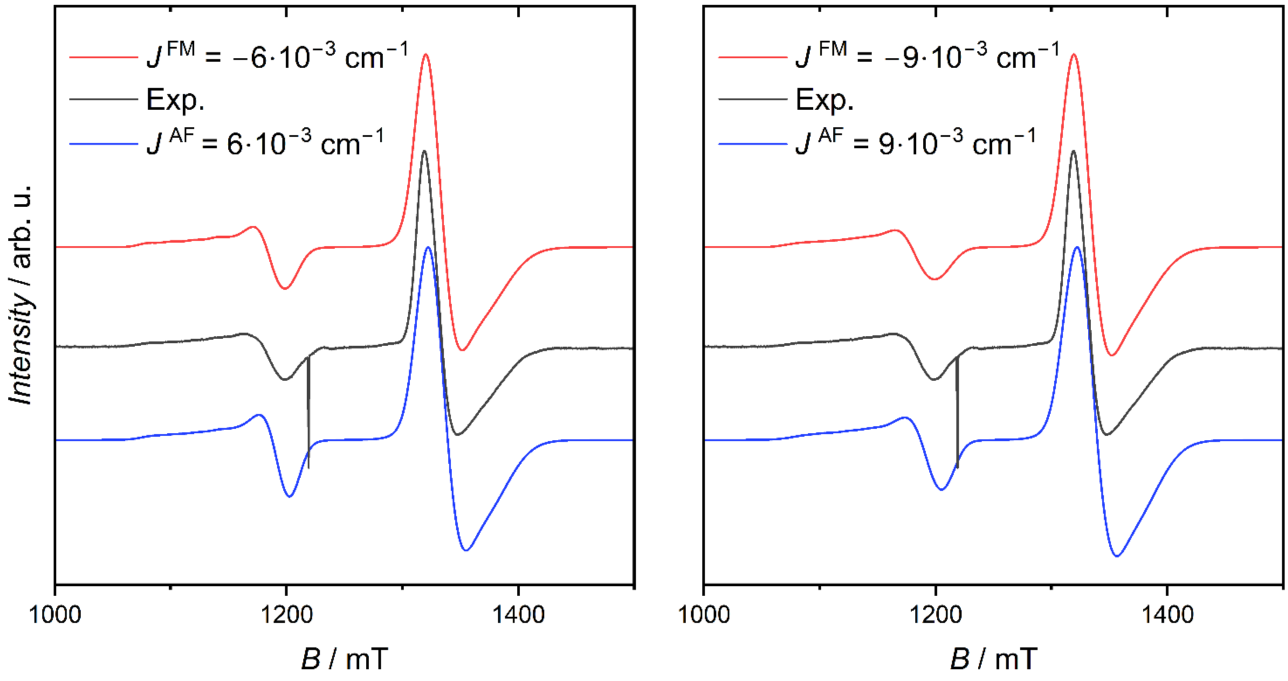

Figure S10. Comparison between simulated spectra obtained by considering FM and AF exchange coupling interaction in **4Cu**. In this case, g-strain components were considered as [0.03 0.03 0.03]. Experimental Q-band EPR spectrum of **4Cu** 0.5mM solutions in 1:1 CH_2Cl_2/toluene at 5 K is reported in the same plots.

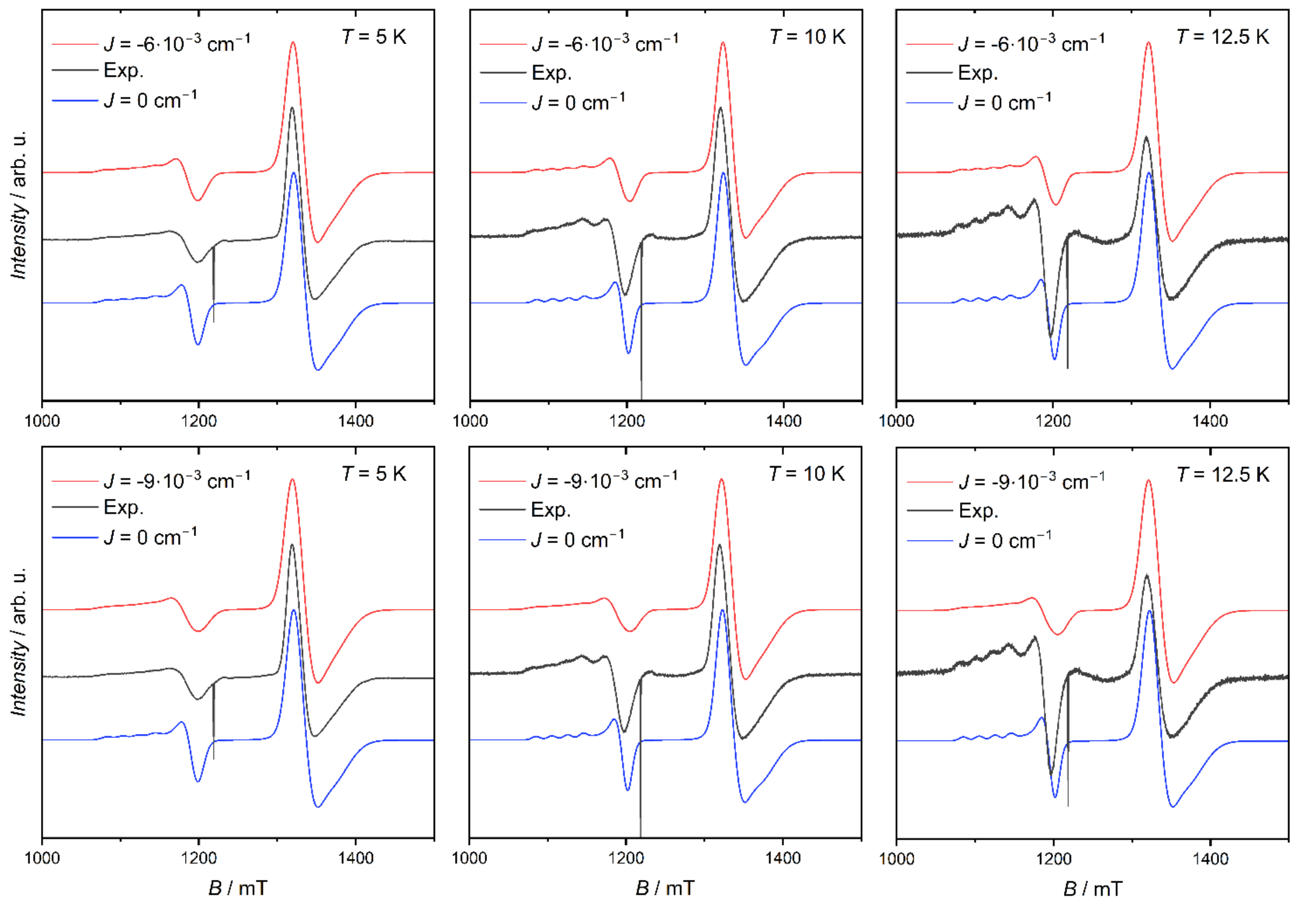

Figure S11. Plot of simulated and experimental spectra for **4Cu** 0.5mM solutions in 1:1 CH_2Cl_2/toluene at 5 K. On top, simulation was performed by considering FM exchange coupling interaction between Cu and {Cr_7Ni} centers. The same strain values reported in Table S4 were used either when exchange coupling interaction is considered or in those with J set to zero.

Table S4. SH parameters for **4Cu** (Figures S11). The same values of $\boldsymbol{g}$ and $\boldsymbol{A}$ components were used for all the other simulations reported in Figures S8 – S10. $^{a}10^{-4}$ cm^{-1}. ^{b} g-strain (FWHM) components.

	T(K)	g_x	g_y	g_z	$A_x{}^a$	$A_y{}^a$	$A_z{}^a$	$\Gamma_x{}^b$ (10^{-3})	$\Gamma_y{}^b$ (10^{-3})	$\Gamma_z{}^b$ (10^{-3})
**Cu**	5	2.038(1)	2.038(1)	2.184(1)	27(2)	27(2)	200(2)	30(2)	30(2)	30(2)
	10	2.038(1)	2.038(1)	2.184(1)	27(2)	27(2)	200(2)	20(2)	20(2)	20(2)
	12.5	2.038(1)	2.038(1)	2.184(1)	27(2)	27(2)	200(2)	20(2)	20(2)	20(2)
**Cr_7Ni**	5	1.843(1)	1.830(1)	1.778(1)				25(1)	30(1)	60(2)
	10	1.843(1)	1.830(1)	1.778(1)				25(1)	30(1)	60(2)
	12.5	1.843(1)	1.830(1)	1.778(1)				25(1)	30(1)	60(2)

S.4 EASYSPIN codes

Here, we report the spin-system core of EASYSPIN [9] codes employed for simulations. We only report codes for simulations at 5 K for simplicity.

1VO

```
%Spin System
Sys.S = [1/2 1/2];
Sys.g = [1.979 1.979 1.958;1.843 1.830 1.778];
Sys.gFrame = [0 0 0;0 90 0]*pi/180;
Sys.Nucs = '51V, 58Ni;
Sys.A = [56 56 161 0 0 0; 0 0 0 0 0 0]*clight*1e-8;
Sys.AFrame = [0 0 0 0 0 0; 0 0 0 0 90 0]*pi/180;

%Magnetic Isotropic Exchange Interactions
Sys.J = -0.0065*clight*1e-4;

%Magnetic Dipolar Interaction
Sys.eeD = [0.0017 -0.0034 0.0017]*clight*1e-4;

%Line Broadening
Sys.lwpp = [0.5 1.0];
Sys.HStrain = [30 30 40];
Sys.gStrain = [0.01 0.01 0.009; 0.015 0.020 0.050];
```

4VO

```
%Spin System
Sys.S = [1/2 1/2 1/2 1/2 1/2];
Sys.g = [1.979 1.979 1.958;1.843 1.830 1.778;1.843 1.830 1.778;...
         1.843 1.830 1.778;1.843 1.830 1.778];
Sys.gFrame = [0 0 0;0 90 0;90 90 0;0 -90 0;-90 -90 0]*pi/180;
Sys.Nucs = '51V, 58Ni, 58Ni, 58Ni, 58Ni';
Sys.A = [56 56 161 0 0 0 0 0 0 0 0 0 0 0 0; 0 0 0 0 0 0 0 0 0 0 0 0 0 0 0;...
          0 0 0 0 0 0 0 0 0 0 0 0 0 0 0;0 0 0 0 0 0 0 0 0 0 0 0 0 0 0;...
          0 0 0 0 0 0 0 0 0 0 0 0 0 0 0]*clight*1e-8;
Sys.AFrame = [0 0 0 0 0 0 0 0 0 0 0 0 0 0 0; 0 0 0 0 90 0 0 0 0 0 0 0 0 0 0; ...
               0 0 0 0 0 0 90 -90 0 0 0 0 0 0 0; 0 0 0 0 0 0 0 0 0 0 -90 0 0 0 0;...
               0 0 0 0 0 0 0 0 0 0 0 0 -90 -90 0]*pi/180;

%Magnetic Isotropic Exchange Interactions
J12 = -6.5e-3; %cm-1
J13 = J12;
J14 = J12;
J15 = J12;
J23 = 0;
J24 = J23;
J25 = J23;
J34 = J23;
J35 = J23;
J45 = J23;
Sys.J = [J12 J13 J14 J15 J23 J24 J25 J34 J35 J45]*clight*1e-4;

%Magnetic Dipolar Interaction
```

```
D12 = [-0.0034 0.0017 0.0017]*clight*1e-4;
D13 = [0.0017 -0.0034 0.0017]*clight*1e-4;
D14 = [-0.0034 0.0017 0.0017] *clight*1e-4;
D15 = [0.0017 -0.0034 0.0017] *clight*1e-4;
D23 = [0.0011 -0.0022 0.0011] *clight*1e-4;
D24 = [-0.380 0.188 0.192]*1e-3*clight*1e-4;
D25 = [0.0011 -0.0022 0.0011]*clight*1e-4;
D34 = [-0.0022 0.0011 0.0011] *clight*1e-4;
D35 = [0.192 -0.380 0.188]*1e-3*clight*1e-4;
D45 = [-0.0022 0.0011 0.0011]*clight*1e-4;
Sys.eeD = [D12;D13;D14;D15;D23;D24;D25;D34;D35;D45];

%Line Broadening
Sys.lwpp = [0.5 1.0];
Sys.HStrain = [30 30 40];
Sys.gStrain = [0.01 0.01 0.009; 0.025 0.030 0.060; 0.025 0.030 0.060; ...
               0.025 0.030 0.060;   0.025 0.030 0.060];
```

4Cu

```
%Spin System
Sys.S=[1/2 1/2 1/2 1/2 1/2];
Sys.g=[2.038 2.038 2.184;1.839 1.814 1.765;1.839 1.814 1.765;...
       1.839 1.814 1.765;1.839 1.814 1.765];
Sys.gFrame=[0 0 0;0 90 0;90 90 0;0 -90 0;-90 -90 0]*pi/180;
Sys.Nucs='Cu, 58Ni, 58Ni, 58Ni, 58Ni';
Sys.A=[27 27 200.1 0 0 0 0 0 0 0 0 0 0 0 0; 0 0 0 0 0 0 0 0 0 0 0 0 0 0 0;...
        0 0 0 0 0 0 0 0 0 0 0 0 0 0 0;0 0 0 0 0 0 0 0 0 0 0 0 0 0 0;...
        0 0 0 0 0 0 0 0 0 0 0 0 0 0 0]*clight*1e-8;
Sys.AFrame=[0 0 0 0 0 0 0 0 0 0 0 0 0 0 0; 0 0 0 0 90 0 0 0 0 0 0 0 0 0 0; ...
            0 0 0 0 0 0 90 -90 0 0 0 0 0 0 0; 0 0 0 0 0 0 0 0 0 0 -90 0 0 0 0;...
            0 0 0 0 0 0 0 0 0 0 0 0 -90 -90 0]*pi/180;

%Magnetic Isotropic Exchange Interactions
J12=-6e-3; %cm-1
J13=J12;
J14=J12;
J15=J12;
J23=0;
J24=J23;
J25=J23;
J34=J23;
J35=J23;
J45=J23;
Sys.J = [J12 J13 J14 J15 J23 J24 J25 J34 J35 J45]*clight*1e-4;

%Magnetic Dipolar Interaction
D12=[-0.0036 0.0018 0.0018]*clight*1e-4;
D13=[0.0018 -0.0036 0.0018]*clight*1e-4;
D14=[-0.0036 0.0018 0.0018]*clight*1e-4;
D15=[0.0018 -0.0036 0.0018]*clight*1e-4;
D23=[0.0011 -0.0022 0.0011]*clight*1e-4;
D24=[-0.380 0.188 0.192]*1e-3*clight*1e-4;
D25=[0.0011 -0.0022 0.0011]*clight*1e-4;
D34=[-0.0022 0.0011 0.0011]*clight*1e-4;
D35=[0.192 -0.380 0.188]*1e-3*clight*1e-4;
D45=[-0.0022 0.0011 0.0011]*clight*1e-4;
Sys.eeD = [D12;D13;D14;D15;D23;D24;D25;D34;D35;D45];

%Line Broadening
```

```
Sys.lwpp= [0.5 1.0];
Sys.HStrain = [30 30 40];
Sys.gStrain = [0.03 0.03 0.03; 0.025 0.030 0.060; 0.025 0.030 0.060; 0.025 0.030 0.060;
0.025 0.030 0.060];
```

References

(1) Yamabayashi, T.; Atzori, M.; Tesi, L.; Cosquer, G.; Santanni, F.; Boulon, M.-E.; Morra, E.; Benci, S.; Torre, R.; Chiesa, M.; Sorace, L.; Sessoli, R.; Yamashita, M. Scaling Up Electronic Spin Qubits into a Three-Dimensional Metal–Organic Framework. J Am Chem Soc 2018, 140 (38), 12090–12101. https://doi.org/10.1021/jacs.8b06733.

(2) Kadish, K. M.; Sazou, D.; Araullo, C.; Liu, Y. M.; Saoiabi, A.; Ferhat, M.; Guilard, R. Electrochemistry of Vanadyl Porphyrins in Dimethylformamide. Inorg Chem 1988, 27 (13), 2313–2320.

(3) Barbosa Neto, N. M.; De Boni, L.; Mendonça, C. R.; Misoguti, L.; Queiroz, S. L.;. Dinelli, L. R.; Batista, A. A.; Zilio, S. C. Nonlinear Absorption Dynamics in Tetrapyridyl Metalloporphyrins. J. Phys. Chem. B 2005, 109 (36), 17340–17345. https://doi.org/10.1021/jp052168i

(4) Timco, G. A.; McInnes, E. J. L.; Pritchard, R. G.; Tuna, F.; Winpenny, R. E. P. Heterometallic Rings Made From Chromium Stick Together Easily. Angew. Chem. Int. Ed. 2008, 47 (50), 9681–9684. https://doi.org/10.1002/anie.200803637.

(5) Rigaku Oxford Diffraction, (2024), CrysAlisPro Software system, version 1.171.42a, Rigaku Corporation, Wroclaw, Poland.

(6) G. M. Sheldrick, SHELXT – Integrated space-group and crystal-structure determination. Acta. Cryst. 2015, A71, 3–8. https://doi.org/10.1107/S2053273314026370

(7) G. M. Sheldrick, Crystal structure refinement with SHELXL. Acta. Cryst. 2015, C71, 3–8. https://doi.org/10.1107/S2053229614024218

(8) O. V. Dolomanov, L. J. Bourhis, R. J. Gildea, J. A. K. Howard, H. Puschmann, *OLEX2*: a complete structure solution, refinement and analysis program. J. Appl. Cryst. 2009, 42, 339–341. https://doi.org/10.1107/S0021889808042726

(9) Stoll, S.; Schweiger, A. EasySpin, a Comprehensive Software Package for Spectral Simulation and Analysis in EPR. Journal of Magnetic Resonance 2006, 178 (1), 42–55. https://doi.org/10.1016/j.jmr.2005.08.013.
